# I-TASSER server: new development for protein structure and function predictions

**DOI:** 10.1093/nar/gkv342

**Published:** 2015-04-16

**Authors:** Jianyi Yang, Yang Zhang

**Affiliations:** 1 Department of Computational Medicine and Bioinformatics, University of Michigan, 100 Washtenaw Avenue, Ann Arbor, MI 48109-2218, USA; 2 School of Mathematical Sciences and LPMC, Nankai University, Tianjin, 300071, PR China; 3 Department of Biological Chemistry, University of Michigan, 100 Washtenaw Avenue, Ann Arbor, MI 48109-2218, USA

## Abstract

The I-TASSER server (http://zhanglab.ccmb.med.umich.edu/I-TASSER) is an online resource for automated protein structure prediction and structure-based function annotation. In I-TASSER, structural templates are first recognized from the PDB using multiple threading alignment approaches. Full-length structure models are then constructed by iterative fragment assembly simulations. The functional insights are finally derived by matching the predicted structure models with known proteins in the function databases. Although the server has been widely used for various biological and biomedical investigations, numerous comments and suggestions have been reported from the user community. In this article, we summarize recent developments on the I-TASSER server, which were designed to address the requirements from the user community and to increase the accuracy of modeling predictions. Focuses have been made on the introduction of new methods for atomic-level structure refinement, local structure quality estimation and biological function annotations. We expect that these new developments will improve the quality of the I-TASSER server and further facilitate its use by the community for high-resolution structure and function prediction.

## INTRODUCTION

With the progress in protein structure prediction, it has become routine for molecular and cytological researchers to seek automated server predictions for their proteins before conducting experimental investigations. The community-wide blind CASP experiments have shown that many automated servers, such as I-TASSER (1,2), Rosetta (3) and HHpred (4), can now generate structural models with accuracy comparable to the best human-expert guided modeling (5–7). Starting from the computational structure modeling, a number of methods have been proposed to annotate the biological function of protein molecules, with examples including COFACTROR (8,9), COACH (10), ConCavity (11), FINDSITE (12), Firestar (13) and 3DLigandSite (14), among many others. A live-bench based platform has been recently embarked to assess the ligand-binding site prediction (15), which demonstrated the considerable accuracy and usefulness of automated protein function annotations (16).

The I-TASSER server represents one of the most widely used online systems for automated protein structure prediction and structure-based function annotation (1,17). Starting from the amino acid sequence, I-TASSER constructs 3D structural models by reassembling fragments excised from threading templates, where the biological insights of the target proteins are deduced by matching the structure models to known proteins in the functional databases (18). Since its first incarnation in 2008 (17), the I-TASSER server system has generated full-length structure models and functional predictions for more than 200 000 proteins submitted by 50 000+ users from 117 countries.

Despite the popular use, we have received numerous comments and suggestions from the user community, on ways to improve the system. In this article, we report on the recent developments made to the I-TASSER server, which have dramatically improved the quality of the I-TASSER modeling and the functionality of the server system. The major new developments include: (i) a new approach in estimating residue-level local quality of the structural models, which are critical to guide functional studies by the biologist users; (ii) an algorithm for B-factor prediction; (iii) methods for atomic-level structure refinement to improve the hydrogen-bonding networks and physical realism of the I-TASSER models; (iv) a consensus-based ligand-binding site prediction that combines structure and sequence profile comparisons by COACH (10); (v) an integration of the new function library BioLiP (18) to increase the coverage of protein functional annotations; and (vi) development of the new message board system for facilitating discussion and communication with the user community.

## MATERIALS AND METHODS

### Overview of the I-TASSER pipeline

**Figure 1.**
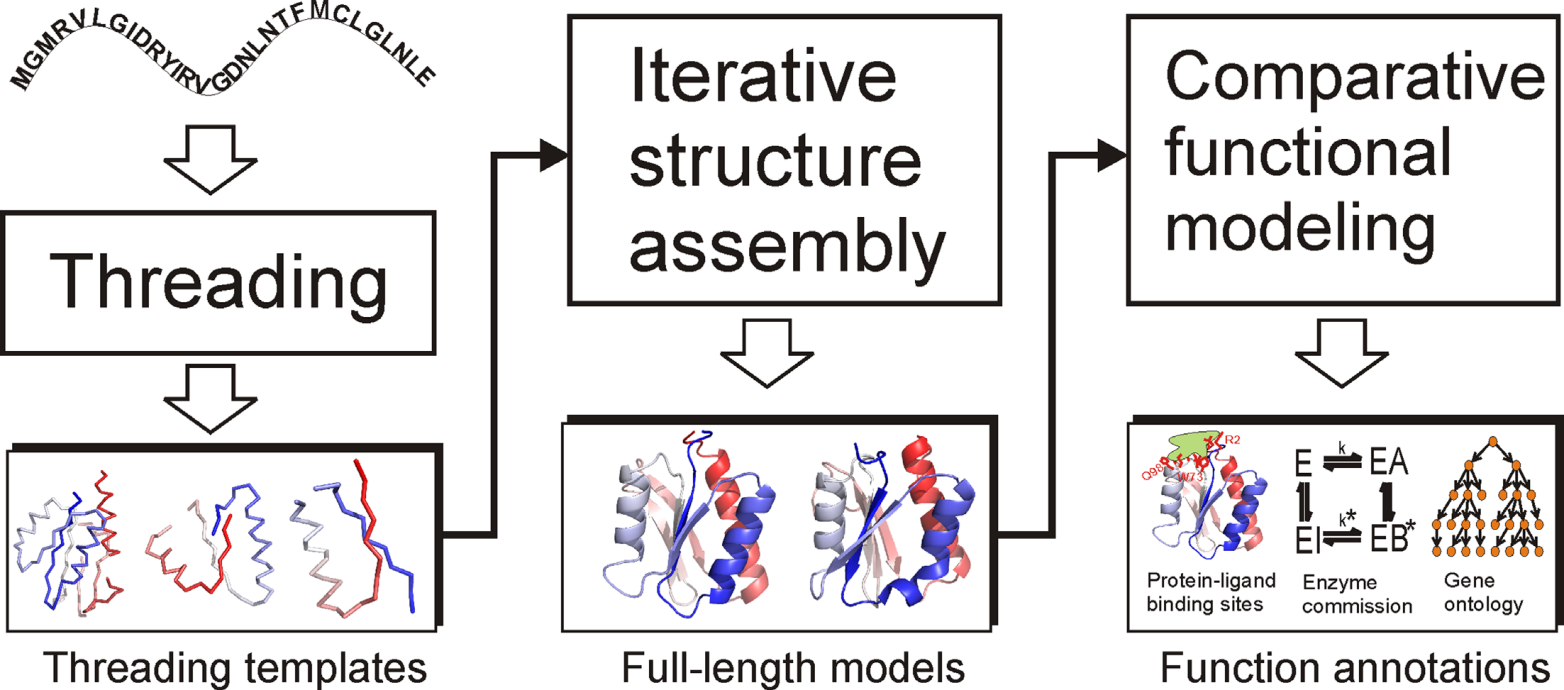
The flowchart of the I-TASSER server pipeline consists of three steps: template identification, full-length structure assembly and structure-based function annotation.

The I-TASSER server (http://zhanglab.ccmb.med.umich.edu/I-TASSER/) is built on I-TASSER, a hierarchical template-based method for protein structure and function predictions consisting of three general steps (Figure 1). For a given query sequence, I-TASSER first identifies structural templates or super secondary structure motifs from the PDB library (19) using LOMETS (20), a meta-threading program that consists of multiple threading algorithms. The topology of the full-length models is then constructed by reassembling the continuously-aligned fragment structures excised from the templates, where the structures of the unaligned regions are built from scratch by *ab initio* folding based on replica-exchange Monte Carlo simulations (21). The structure trajectories are clustered by SPICKER (22) to identify low free-energy states. Starting from the SPICKER clusters, a second round of structure reassembly is conducted to refine the structural models. The low free-energy conformations are further refined by full-atomic simulations using FG-MD (23) and ModRefiner (24). Finally, functional insights of the query protein are obtained by matching the structural model with proteins in the BioLiP function library via structure and sequence profile comparisons (8,10,18).

The I-TASSER pipeline is identical to the approach used by Zhang-Server in the CASP experiments. Since CASP9, however, a new *ab initio* structure prediction approach, QUARK (25), has been introduced to the Zhang-Server pipeline to recognize and sort templates for the hard free modeling (FM) targets (26,27). The QUARK program has not yet been integrated into the I-TASSER server, which is available as an independent online server for *ab initio* folding targets at http://zhanglab.ccmb.med.umich.edu/QUARK/.

### Recent developments of the I-TASSER server

#### New approach to estimating residue-level local quality of I-TASSER models

I-TASSER provides a confidence score (C-score) to estimate the models’ global accuracy. However, the residue-level local accuracy, which is critical for structure-based functional studies such as active site recognition, ligand–protein docking and drug screening, was missed in previous I-TASSER structure prediction pipelines. To address this issue, we have developed a new algorithm, ResQ (Yang *et al*., submitted), for estimating the residue-specific local quality of the I-TASSER models. Multiple sources of information are considered in ResQ, including: (i) the structural variations of Monte Carlo assembly simulations, (ii) distance deviations of the templates detected by threading and structural alignment, (iii) the threading alignment coverage and (iv) the consistency between the model and the sequence-based predictions of structural features.

The residue-specific quality estimation by ResQ was assessed on a non-redundant set of 635 proteins with their structure models generated by I-TASSER, where the average difference between the estimated and observed distance errors of the I-TASSER models is 1.4 Å for the 506 proteins with C-score > −1.5. The ResQ method was also tested with the structure models generated by a variety of predictors in CASP9 and CASP10 experiments. The results showed that ResQ is comparable to, or outperforms most of, the model quality assessment programs (MQAPs) for the local structure quality estimation (see Supplementary Tables S1–S4). The ResQ program can be used to assess the accuracy of structure models generated by both I-TASSER and other structure prediction methods. It can be accessed through an online server at http://zhanglab.ccmb.med.umich.edu/ResQ/ or as a standalone package downloadable through the I-TASSER Suite at http://zhanglab.ccmb.med.umich.edu/I-TASSER/download.

#### B-factor prediction

Another highly-relevant but often-missed local feature in the structure prediction pipelines is the inherent thermal mobility of residues in proteins. At the absolute zero temperature, the atoms in a protein are assumed to stay at the equilibrium position of lowest energy; but as the temperature increases, the ambient thermal energy causes the atoms to oscillate around the equilibrium position, the extent of which often varies depending on the relative location on the 3D structure and the interaction with ligand and solvent atoms. The atomic motion can be experimentally measured in X-ray crystallography as a B-factor (or temperature factor), which was introduced as an amendment factor to the structure factor equation, since the scattering effect of X-rays is reduced on the oscillating atoms compared to the atoms at rest (28). Because the distribution of the thermal motion factors in protein crystals can be affected by systematic errors, such as experimental resolution, crystal contacts and refinement procedures, the raw B-factor values are usually not comparable between different experimental structures. Therefore, to reduce the influence, we calculate a normalized B-factor with a Z-score-based transformation. The normalized B-factor was predicted in ResQ using a combination of template-based assignment and machine learning-based prediction that employs sequence profiling and predicted structural features (Yang *et al*., submitted). Tests on the 635 proteins mentioned above show that the estimated B-factor by ResQ has an average area under the curve (AUC) of 0.79, compared to the X-ray crystallography data (see Supplementary Table S5).

#### Atomic level structure refinement

One of the most frequent complaints in the previous I-TASSER modeling version is the local structure quality of the structural models. For instance, some models have unrealistic secondary structure features or steric clashes, even though the global topology is generally correct. This often happens for non-homologous protein targets where the structural assembly simulations tend to generate divergent decoys due to inconsistent threading alignments. Therefore, the combined structure models from the diverged decoy structures result in distorted local structure motifs.

To improve the local structure quality, we developed two algorithms, FG-MD (23) and ModRefiner (24). In FG-MD (23), we relax the I-TASSER models by simulated annealing molecular dynamics simulations, where the distance maps from structure fragments detected by TM-align from the PDB library are used as spatial restraints. In ModRefiner (24), the structure models are refined with two steps: the backbone structure is first constructed from Cα traces by hydrogen-bonding optimization, then the side-chain rotamers are repacked by quick Monte Carlo simulations using a physics-based atomic force field. The two methods are integrated into the I-TASSER pipeline to refine the models of different targets. In general, FG-MD is used for easy targets with homologous templates; and ModRefiner is employed for hard targets without homologous templates to generate full-atom models that are followed by FG-MD refinements. The data from benchmark and CASP tests have shown that the HB-score (measuring the quality of hydrogen-bonding network) and the Molprobity score (measuring the steric clash and Ramachandran regularity) of the I-TASSER models can be significantly improved by the refinement simulations (27,29).

#### Consensus approach to ligand-binding site prediction

The ligand-binding site in I-TASSER was previously predicted by COFACTOR, which deduces binding sites from homologous templates detected by global and local structure comparisons (8). However, we found that having a method based on structure-based comparisons alone could result in the missing of some useful functional templates due to evolution. As a result, we developed two new methods, one based on binding-specific substructure comparison (TM-SITE) and another on sequence profile alignment (S-SITE), for complementary binding site predictions. When combining TM-SITE and S-SITE with COFACTOR, ConCavity (11) and FINDSITE (12), a consensus approach called COACH was developed, which significantly improved any of the individual methods in our large-scale benchmark tests (10). We believe that the incorporation of other complementary ligand-binding site prediction methods, such as Firestar (13) and 3DLigandSite (14), would further improve the performance of the COACH method.

##### Integration of the new function library BioLiP

I-TASSER annotates the biological function of the query protein, including ligand-binding sites, enzyme commission (EC) number and gene ontology (GO) terms, based on the matching of the structure models with the templates in a function library. The PDB library (19) is the major informational repository for studies involving protein structure and function. But many proteins in the PDB contain redundant entries, incorrectly ordered residues and ambiguous functional information; in particular, many proteins were solved using artificial molecules as additives to facilitate the structural determination. These problems prevent the PDB from being a reliable resource for precise structure-based function analyses. Efforts have been made to address these issues to build biologically-relevant ligand–protein binding databases, such as FireDB (30).

We proposed a hierarchical procedure consisting of four steps of computer-based filtering and manual literature validation for the assessment of biological relevance for each PDB entry. Consequently, the procedure was used to construct a comprehensive function database, BioLiP (18), from databases of known protein structure/function and the literature in PubMed. In addition to the ligand-binding sites and associated binding affinity data, each entry in BioLiP contains EC numbers and GO terms, which are all updated weekly and freely available for the community at http://zhanglab.ccmb.med.umich.edu/BioLiP.

The current release of BioLiP (Jan 16, 2015) contains 304 050 entries constructed from 65 506 PDB proteins, in which 37 685 entries are for DNA/RNA–protein interactions, 13 967 for peptide–protein interactions, 85 652 for metal ion–protein interactions and 166 746 for regular small molecule–protein interactions. It involves 76 881 protein chains with 518 and 2447 unique first three-digit and four-digit EC numbers, respectively, and 37 178 chains with known catalytic sites. It also contains 119 004 chains associated with 8315 unique GO terms. These data provide a comprehensive resource for structure-based function annotation in the I-TASSER server.

#### Development of the new message board system

We received numerous questions from the users through emails, letters and phone calls about the server systems developed in our lab. To facilitate communication among the users and/or between the user community and the lab members, a discussion board system was established at http://zhanglab.ccmb.med.umich.edu/bbs. This helps initiate discussion between the users, as well as enables quick response to the users’ problems in using the programs and/or interpreting the modeling results by our lab members. The message board also helps us to collect feedback from users, which are important in helping us improve our algorithms and services for better serving the community. There are currently ∼3000–4000 daily accesses to the board from the user community. Since its launch in 2011, we have received and answered >1500 comments/threads on our services from more than 2500 registered users, which shows that the message board is an effective forum for user engagement and an excellent source for service improvement.

## INPUT AND OUTPUT OF THE SERVER

### Input

The input to the I-TASSER server is the primary amino acid sequence of the query protein. The server provides three additional options for advanced users to input more information to guide the I-TASSER modeling. The first option is for specifying distance constraints and structure templates to assist in modeling. The second one is for excluding some templates from the I-TASSER template library, designed for some special purposes (e.g. for benchmark test). The third one allows users to specify secondary structure for specific residues. For a protein with ∼400 residues, it takes 10–24 h for the server to generate the complete set of modeling results.

### Output

For each submission, one unique job ID and one URL are assigned to track its modeling status. The user will be notified by email when the modeling has completed, and the resulting data are reported on a webpage at the URL assigned. An example output page is available at: http://zhanglab.ccmb.med.umich.edu/I-TASSER/example. The output data include: (i) a summary of the submitted sequence and local structural feature prediction, (ii) the top 10 threading templates used, (iii) the top-ranked 3D structure models with global and local accuracy estimations, (iv) the top 10 proteins with similar structures to the query and (v) structure-based function annotations on ligand-binding site, EC number and GO terms. The modeling results are kept on the server for 90 days and will be deleted after that to save disc space in our system. All the modeling results listed on the result page are collected together in a tarball file, which is provided for download on the same page. Users are encouraged to download this file to their computer to store the results permanently. The output results are introduced briefly below. A graphical explanation of the I-TASSER output is provided at the results annotation page: http://zhanglab.ccmb.med.umich.edu/I-TASSER/annotation.

#### Submitted sequence and predicted structural features

**Figure 2.**
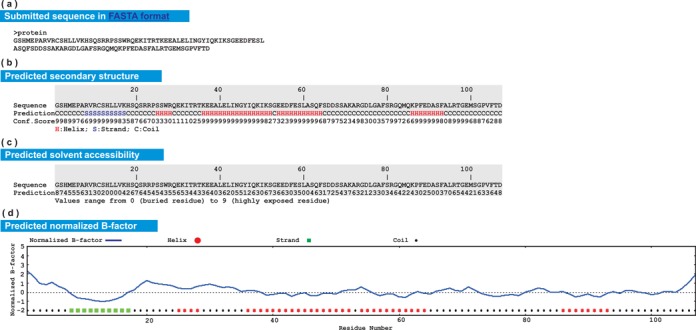
Illustration of submitted sequence and predicted local structure features in the I-TASSER server output. (**a**) Amino acid sequence in FASTA format; (**b**) predicted secondary structure; (**c**) predicted solvent accessibility; (**d**) predicted normalized B-factor.

The first four sections of the I-TASSER result page summarize the submitted amino acid sequence and the predicted local structure features including secondary structure, solvent accessibility and normalized B-factor, which are illustrated in Figure 2. In general, positive B-factor values indicate that the residues are more flexible in the structure, while negative values suggest that the residues are relatively more stable. The predicted secondary structure is also shown in the B-factor plot. Residues located in loop or tail regions tend to have higher predicted B-factor values, as they are usually less stable compared with residues located at other regular secondary structure regions. The user-specified restraints, including template alignments and secondary structure restraints, are also listed in these sections when provided.

#### Top 10 templates used by I-TASSER

With the current set of 14 threading programs in LOMETS (20), up to 140 templates are used by I-TASSER to extract distance restraints. However, the top 10 templates ranked by LOMETS are the most relevant ones because they are given a higher weight in restraints collection and are used as the starting models in the low-temperature replicas in replica-exchange Monte Carlo simulations. The information of these templates is listed in the fifth section of the results page, which includes: (i) the template PDB IDs, (ii) normalized threading Z-scores, (iii) coverage of alignments, (iv) sequence identities and (v) alignments between the query and the templates. While the Z-score corresponds to the difference between the raw alignment score and the mean in units of standard deviation, a normalized Z-score is defined as the Z-score divided by the program-specific Z-score cutoff. Thus, a normalized Z-score >1 indicates a confident alignment. The query protein is classified as an ‘Easy’ target if there are on average at least one template per threading program having the normalized Z-score >1; otherwise, it is considered a ‘Hard’ target.

#### Top five models predicted by I-TASSER

**Figure 3.**
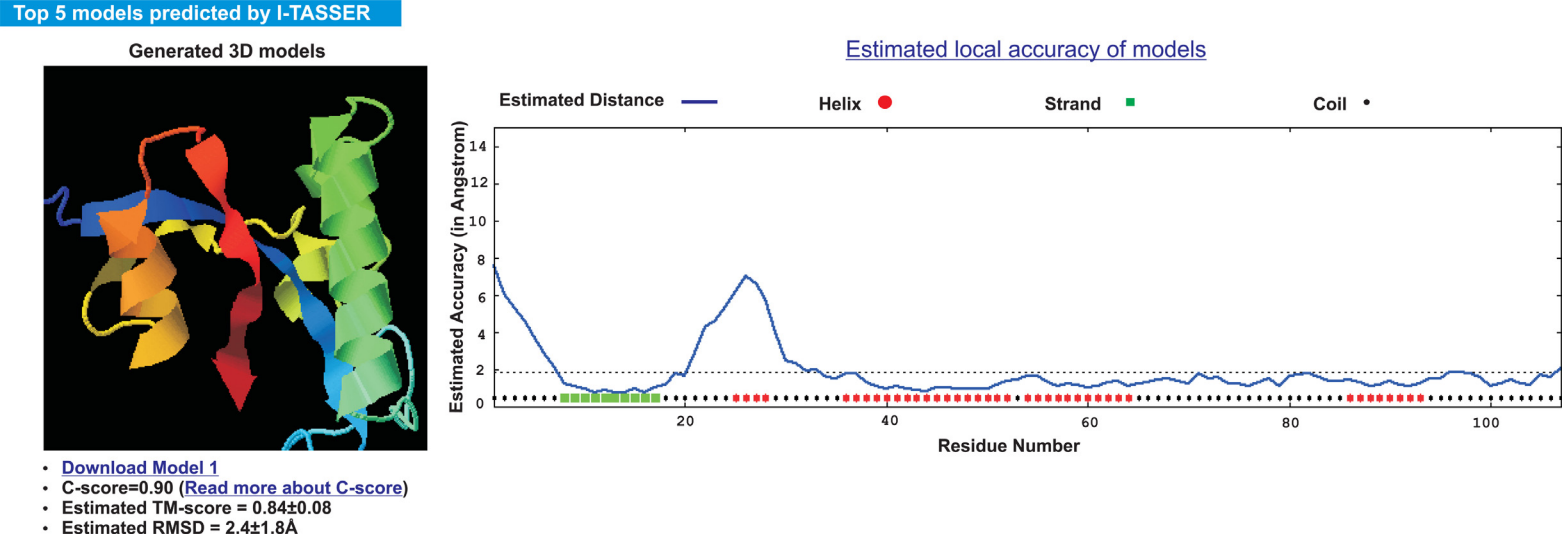
An excerpt of the predicted structure model with global and local accuracy estimations. The structure is visualized in rainbow cartoon by the JSmol applet on the left panel. The estimated local accuracy is shown as a plot on the right panel, which indicates that the N-terminal and the residues between 20 and 30 have relatively higher modeling error while most of other regions are accurate with estimated distance to native smaller than 2 Å in this example.

Up to five full-length structural models, together with the estimated global and local accuracy, are reported in the sixth section of the result page. In the event that the modeling simulations converge, there may be less than five models reported, which is usually an indication that the models have a relatively high confidence. Figure 3 shows the first I-TASSER model of an example protein; it has a global C-score of 0.9, and the estimated TM-score and RMSD are 0.84 and 2.4 Å, respectively. Users can download the PDB-formatted structure file of the model to their own computers in order to visualize the structure locally. The data file for the residue-specific local accuracy estimation and the predicted B-factor values are also available for download by clicking the link ‘Estimated local accuracy of models’ provided on the webpage.

#### Structure analogs in PDB

**Figure 4.**
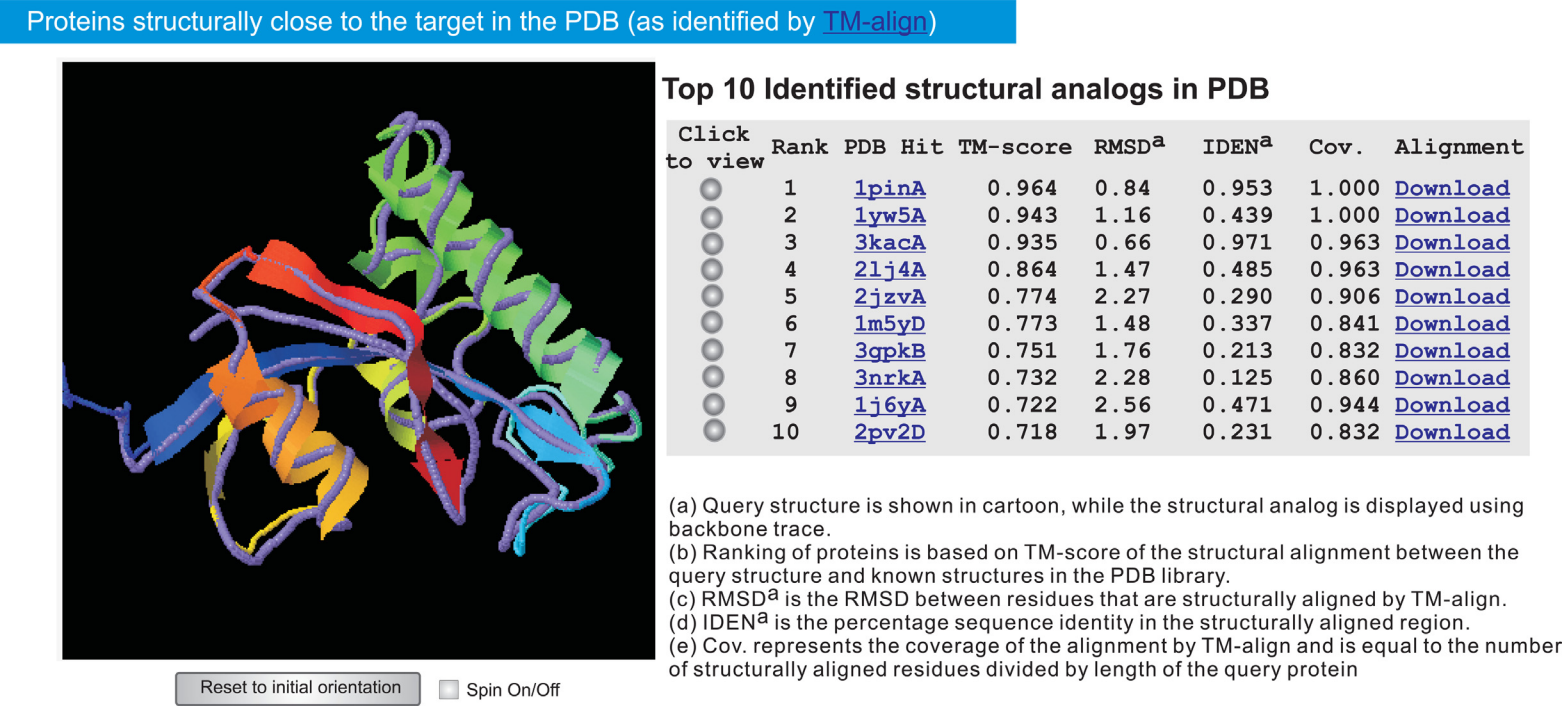
The top 10 PDB proteins that are structurally close to the example protein. The query structure and the PDB proteins are shown in cartoon and backbone, respectively. Each of the structural alignments can be visualized interactively by clicking the corresponding radio buttons.

The first I-TASSER model is searched against the PDB library by TM-align (31) to find the analogs that are structurally similar to the query proteins. Figure 4 shows an example of the searching results. The structural alignments between the query and the 10 closest proteins are ranked by TM-score (32). The table provides the numerical details of the structural alignments, including the TM-score, alignment coverage, RMSD and the sequence identity in the structurally aligned region. The links for downloading the coordinate files of the superimposed structures are provided in the same table. Note that the proteins listed in this section can be different from those listed at the section ‘Top 10 threading templates used by I-TASSER’ because they are detected by different methods. The former is detected by structural alignment based on the first I-TASSER model, while the latter is found by threading from query sequence.

#### Structure-based function annotation by COACH

**Figure 5.**
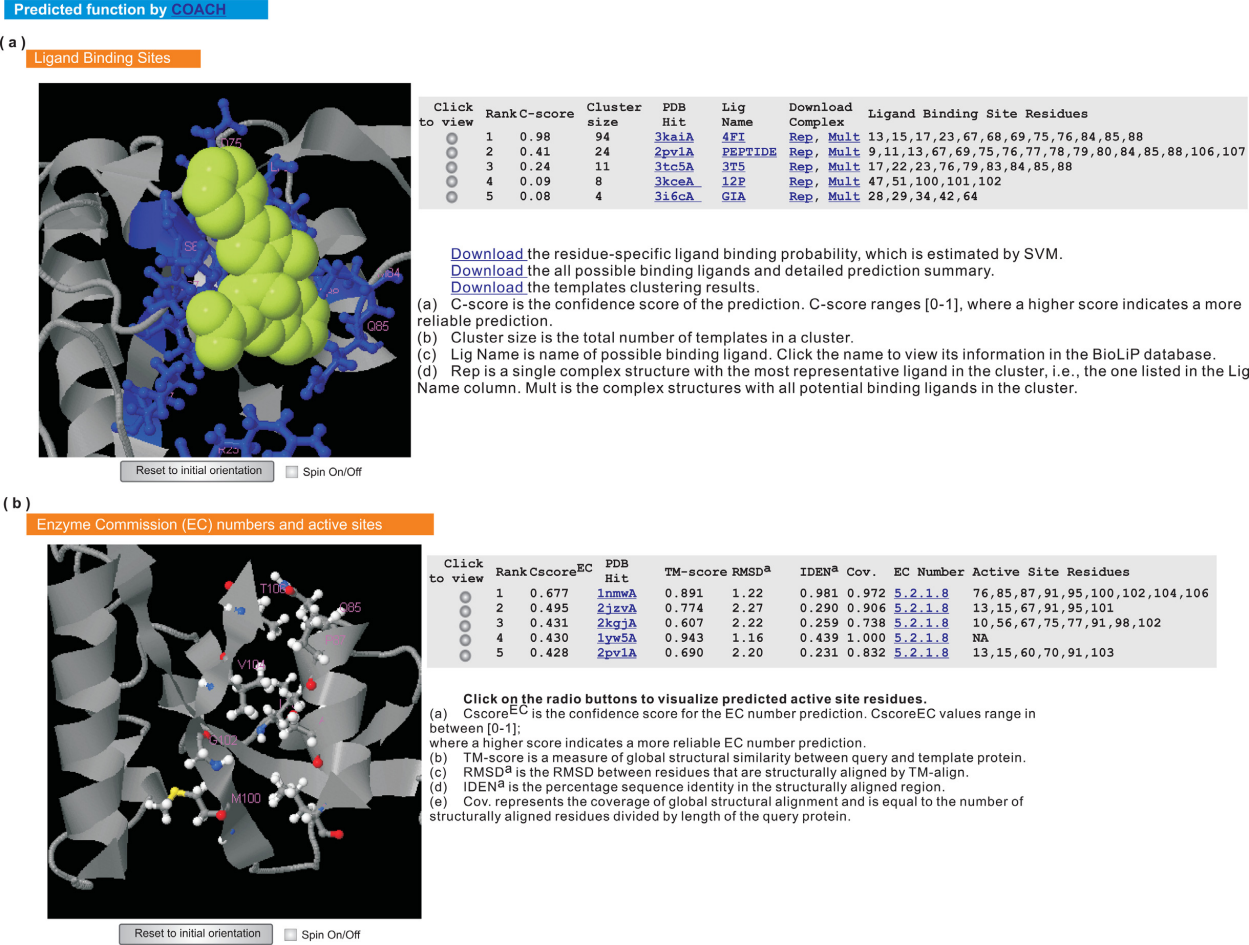
Illustration of the predicted ligand-binding site, enzyme commission number and active site. The query structure is shown in gray cartoon. (**a**) The predicted ligand-binding site. The predicted binding ligands and ligand-binding residues are highlighted in yellow-green spheres and blue ball-and-sticks, respectively. For each prediction, two types of complex structures are provided for download, one containing a representative ligand (i.e. the ‘Rep’ link) and the other containing multiple ligands (i.e., the ‘Mult’ link), respectively. (**b**) The predicted EC number and active site residues are shown in colored ball-and-sticks.

The first I-TASSER model, which in general has the highest confidence score, is submitted to COACH (10) to predict its biological function, including ligand-binding site, EC number and GO terms. An illustrative example of the predicted ligand-binding site, EC number and active site is demonstrated in Figure 5. The predicted GO terms are available in the last section of the results page, which are presented in two parts. The first part lists the top 10 ranked template proteins that are annotated with GO terms. As the template proteins may have additional functional domains, the most frequently-occurring GO terms in each of the three functional aspects (molecular function, biological process and cellular component) are reconciled from the top five homologs, with the resulting consensus GO terms presented in the second part.

## PERFORMANCE OF THE SERVER

I-TASSER-based algorithms have been extensively tested in both benchmark (8,10,23,33) and blind tests (26,27,29,34). For the blind tests, it has participated in the community-wide CASP (35) and CAMEO (15) experiments for protein structure and function predictions. I-TASSER (with the group name ‘Zhang-Server’) was ranked as the top server for automated protein structure prediction in the 7^th^-11^th^ CASP competitions (26,27,29,34). In CAMEO (15), COACH generated ligand-binding site predictions for 4570 targets (between 2012-12-07 and 2015-01-09) with an average AUC score 0.84, which was 18% higher than the second best method in the experiment. These data suggest that the I-TASSER server represents one of the cutting-edge algorithms for automated protein structure and function prediction.

## CONCLUSIONS

The I-TASSER server is an online facility designed for automated protein structure prediction and structure-based function annotation. The involved algorithms have been evaluated rigorously in community-wide blind experiments and demonstrated considerable advantages compared to peer methods in protein structure and function prediction. With numerous feedback from the user community, a variety of new developments have been made to the server to improve the quality of the server in atomic-level structure refinement, structure-based function annotation, local quality estimation and user interface communication.

The template libraries of the server system for both structure and function predictions are updated weekly and free to download on the I-TASSER homepage. Users are encouraged to report and discuss the I-TASSER server-related questions at our message board. We believe that with the help from the user community and the continuous method development, the I-TASSER server is on its way toward the goal of providing the most accurate and useful structure and function predictions based on the state-of-the-art methods.

## SUPPLEMENTARY DATA

Supplementary Data are available at NAR Online.

SUPPLEMENTARY DATA

## References

[1] Roy A., Kucukural A., Zhang Y. (2010). I-TASSER: a unified platform for automated protein structure and function prediction. Nat. Protoc..

[2] Yang J., Yan R., Roy A., Xu D., Poisson J., Zhang Y. (2015). The I-TASSER Suite: protein structure and function prediction. Nat. Methods.

[3] Rohl C.A., Strauss C.E., Misura K.M., Baker D. (2004). Protein structure prediction using Rosetta. Methods Enzymol..

[4] Soding J., Biegert A., Lupas A.N. (2005). The HHpred interactive server for protein homology detection and structure prediction. Nucleic Acids Res..

[5] Battey J.N., Kopp J., Bordoli L., Read R.J., Clarke N.D., Schwede T. (2007). Automated server predictions in CASP7. Proteins.

[6] Huang Y.J., Mao B., Aramini J.M., Montelione G.T. (2014). Assessment of template-based protein structure predictions in CASP10. Proteins.

[7] Tai C.H., Bai H., Taylor T.J., Lee B. (2014). Assessment of template-free modeling in CASP10 and ROLL. Proteins.

[8] Roy A., Yang J., Zhang Y. (2012). COFACTOR: an accurate comparative algorithm for structure-based protein function annotation. Nucleic Acids Res..

[9] Roy A., Zhang Y. (2012). Recognizing protein-ligand binding sites by global structural alignment and local geometry refinement. Structure.

[10] Yang J., Roy A., Zhang Y. (2013). Protein-ligand binding site recognition using complementary binding-specific substructure comparison and sequence profile alignment. Bioinformatics.

[11] Capra J.A., Laskowski R.A., Thornton J.M., Singh M., Funkhouser T.A. (2009). Predicting protein ligand binding sites by combining evolutionary sequence conservation and 3D structure. PLoS Comput. Biol..

[12] Brylinski M., Skolnick J. (2008). A threading-based method (FINDSITE) for ligand-binding site prediction and functional annotation. Proc. Natl. Acad. Sci. U.S.A..

[13] Lopez G., Maietta P., Rodriguez J.M., Valencia A., Tress M.L. (2011). firestar–advances in the prediction of functionally important residues. Nucleic Acids Res..

[14] Wass M.N., Kelley L.A., Sternberg M.J. (2010). 3DLigandSite: predicting ligand-binding sites using similar structures. Nucleic Acids Res..

[15] Haas J., Roth S., Arnold K., Kiefer F., Schmidt T., Bordoli L., Schwede T. (2013). The Protein Model Portal–a comprehensive resource for protein structure and model information. Database (Oxford).

[16] Zhang Y. (2009). Protein structure prediction: when is it useful. Curr. Opin. Struct. Biol..

[17] Zhang Y. (2008). I-TASSER server for protein 3D structure prediction. BMC Bioinformatics.

[18] Yang J., Roy A., Zhang Y. (2013). BioLiP: a semi-manually curated database for biologically relevant ligand-protein interactions. Nucleic Acids Res..

[19] Berman H.M., Westbrook J., Feng Z., Gilliland G., Bhat T.N., Weissig H., Shindyalov I.N., Bourne P.E. (2000). The Protein Data Bank. Nucleic Acids Res..

[20] Wu S., Zhang Y. (2007). LOMETS: a local meta-threading-server for protein structure prediction. Nucleic Acids Res..

[21] Zhang Y., Kolinski A., Skolnick J. (2003). TOUCHSTONE II: a new approach to ab initio protein structure prediction. Biophys. J..

[22] Zhang Y., Skolnick J. (2004). SPICKER: a clustering approach to identify near-native protein folds. J. Comput. Chem..

[23] Zhang J., Liang Y., Zhang Y. (2011). Atomic-level protein structure refinement using fragment-guided molecular dynamics conformation sampling. Structure.

[24] Xu D., Zhang Y. (2011). Improving the physical realism and structural accuracy of protein models by a two-step atomic-level energy minimization. Biophys. J..

[25] Xu D., Zhang Y. (2012). Ab initio protein structure assembly using continuous structure fragments and optimized knowledge-based force field. Proteins.

[26] Xu D., Zhang J., Roy A., Zhang Y. (2011). Automated protein structure modeling in CASP9 by I-TASSER pipeline combined with QUARK-based ab initio folding and FG-MD-based structure refinement. Proteins.

[27] Zhang Y. (2014). Interplay of I-TASSER and QUARK for template-based and ab initio protein structure prediction in CASP10. Proteins.

[28] Sherwood D., Cooper J. (2011). Crystals, X-rays and Proteins: Comprehensive Protein Crystallography.

[29] Zhang Y. (2009). I-TASSER: fully automated protein structure prediction in CASP8. Proteins.

[30] Maietta P., Lopez G., Carro A., Pingilley B.J., Leon L.G., Valencia A., Tress M.L. (2014). FireDB: a compendium of biological and pharmacologically relevant ligands. Nucleic Acids Res..

[31] Zhang Y., Skolnick J. (2005). TM-align: a protein structure alignment algorithm based on the TM-score. Nucleic Acids Res..

[32] Zhang Y., Skolnick J. (2004). Scoring function for automated assessment of protein structure template quality. Proteins.

[33] Wu S., Skolnick J., Zhang Y. (2007). Ab initio modeling of small proteins by iterative TASSER simulations. BMC Biol..

[34] Zhang Y. (2007). Template-based modeling and free modeling by I-TASSER in CASP7. Proteins.

[35] Moult J., Fidelis K., Kryshtafovych A., Schwede T., Tramontano A. (2014). Critical assessment of methods of protein structure prediction (CASP)–round x. Proteins.

