# Supplementary material for: I-TASSER server: new development for protein structure and function predictions
Source: Nucleic Acids Res. 2015 Apr 16;43(Web Server issue):W174–81. doi: 10.1093/nar/gkv342 (PMC4489253; doi:10.1093/nar/gkv342)
Supplement: SUPPLEMENTARY DATA [file supp_43_W1_W174__index.html]

I-TASSER server: new development for protein structure and function predictions — SUPPLEMENTARY DATA 

# I-TASSER server: new development for protein structure and function predictions

## SUPPLEMENTARY DATA

**Files in this Data Supplement:**

- SUPPLEMENTARY DATA
