# Supplementary material for: I-TASSER server: new development for protein structure and function predictions
Source: Nucleic Acids Res. 2015 Apr 16;43(Web Server issue):W174–81. doi: 10.1093/nar/gkv342 (PMC4489253; doi:10.1093/nar/gkv342)
Supplement: SUPPLEMENTARY DATA [file supp_gkv342_nar-00222-web-b-2015-File007.pdf]

I-TASSER server: new development for protein structure and function predictions

Jianyi Yang and Yang Zhang

Supplementary Material

This section describes the benchmark test results of the ResQ program, which was designed to quantitatively estimate B-factor and residue-level error in protein structure prediction (1).

Datasets

A total number of 1,270 non-redundant single-domain proteins were collected from the Protein Data Bank (2), which have a pair-wise sequence identity <30% with size ranging from 50 and 300 residues. We randomly selected half of the proteins for training and the remaining for test. I-TASSER was used to generate structure predictions for the 635 testing proteins that are non-redundant to the other 635 proteins used to train ResQ. 483 proteins are categorized into Easy and 152 into Hard targets according to the significance score of the LOMETS alignments (3). After excluding the homologous templates with a sequence identity >30% from the template library, the I-TASSER simulations generated the first models with an average TM-score 0.71 and RMSD 5.7 Å; these modeling results are largely consistent with the results from the I-TASSER modeling in the recent CASP experiments (4,5).

Assessment criteria of the residue-specific quality and B-factor profile predictions

Three measures are used to evaluate the accuracy of the residue-specific quality (RSQ) prediction. The first is the Pearson's correlation coefficient (PCC) between the predicted (d_p) and observed (d_o) distances of the model to the native structure. The second measure is the area under the curve (AUC) of the receiver-operating characteristic (ROC), which is designed to evaluate the ability of ResQ in discriminating between well and badly modeled regions, where a residue is defined as 'well modeled' (positive) if the distance from model to the native is <3.8 Å upon the TM-score superposition, otherwise as 'badly modeled' (or negative). These two metrics were also used by the CASP assessors for evaluating the accuracy of model quality estimation (6). Following Kryshtafovych et al, we converted d_p into the range of (0, 1) by $d_p'=1/[1+(d_p/5)^2]$ in the AUC calculation, so that a fixed number of divisions can be used for different data samples to draw the ROC curves.

The third metric for evaluating the RSQ prediction is the average difference (Δd) between d_p and d_o, i.e.

$$\Delta = \frac{1}{L}\sum_{i=1}^{L}\left|d_o^i - d_p^i\right| \qquad (1)$$

where L is the length of the protein.

The B-factor profile (BFP) prediction is evaluated by the Pearson's correlation between the predicted and the experimental B-factors, which was also used in previous B-factor prediction studies (7,8). Similar to the RSQ evaluation, we also use AUC for measuring the ability in discriminating between

stable and flexible residues in structures, where a residue is defined as stable (positive) if the normalized B-factor is below 0 or as flexible (negative) otherwise. Similarly, for even ROC division we renormalized the predicted B-factor values (b) to the range of (0, 1) by $1/[1+\exp(-b)]$.

Test results of residue-specific quality prediction

ResQ was applied to the first I-TASSER models of the 635 testing proteins to estimate the distance of each residue to the native structure. As shown in Table S1, the average distance predicted by ResQ (d_p) is 3.4 Å, which is consistently lower than the observed distance (d_o) of the residues on the models to that on the native (4.3 Å), resulting in an average difference between d_p and d_o, Δd=2.4 Å. This consistent reduction of distance estimation relative to the native structure is mainly due to the lower distance estimation for the residues of large modeling errors (1).

We further split the test proteins into two groups, following the I-TASSER confidence score (C-score), i.e., the high- and low-confidence groups with a C-score above or below -1.5, a cutoff that was proofed to generate the lowest false positive and false negative rates for the I-TASSER modeling (9). As expected, the I-TASSER models with a higher C-score have a much better quality (TM-score=0.8) than that of a lower C-score (TM-score=0.4). Accordingly, the RSQ prediction for the high C-score proteins is much more accurate (Δd=1.4 Å) than that of low C-score ((Δd=6.4 Å), and the average PCC and AUC are 30% and 14% higher, respectively, for the high C-score models than that for the low C-score models (Table S1).

Table S1. Summary of the RSQ predictions on the 635 test proteins.

**Groups**	**N_{pro}**	**TM**	**d_o**	**d_p**	**Δd**	**PCC**	**AUC**
C-score>-1.5	506	0.80	2.7	2.2	1.4	0.69	0.89
C-score<-1.5	129	0.40	10.8	8.5	6.4	0.53	0.78
Overall	635	0.71	4.3	3.4	2.4	0.66	0.87

N_{pro}: Number of proteins in the set.

TM: Average TM-score of the first I-TASSER model.

$d_o(d_p)$: Observed (predicted) distance between the model and the native structure.

Δd: Difference between d and d' based on Eq. (1).

In Table S2-S4, we also list the results of ResQ on the CASP9 and CASP10 models in comparison with the top-performing model quality assessment programs (MQAPs). These data showed that ResQ outperforms most of the MQAP methods in the local quality estimation of protein structure predictions.

Table S2. Comparison between ResQ and other MQAPs for RSQ prediction on the CASP9 decoys. N_m is the total number of models that a predictor submitted. Best scores are highlighted in bold in each category.

Methods	Δd	PCC	AUC	N_m
ResQ	**3.073**	**0.727**	0.883	25694
QMEANclust	3.600	0.718	0.877	25611
MULTICOM	3.670	0.720	0.872	24799
MULTICOM-REFINE	4.121	0.693	0.859	25694
MULTICOM-CONSTRUCT	4.166	0.655	0.851	24691

MQAPmulti	4.331	0.679	0.870	24587
MetaMQAPclust	4.335	0.695	0.876	25057
MQAPsingle	4.765	0.629	0.843	25057
PconsM	5.145	0.656	0.878	25572
ModFOLDclust2	5.163	0.700	**0.898**	25626
IntFOLD-QA	5.173	0.698	**0.898**	25425

Table S3. Comparison between ResQ and other MQAPs on the Stage-1 of CASP10 MQAP experiment, where all redundant models have been excluded. N_m is the total number of models that a predictor submitted. Best scores are highlighted in bold in each category.

Methods	Δd	PCC	AUC	N_m
ResQ	**4.010**	**0.666**	0.849	1438
MQAPfrag2	4.156	0.633	0.828	1438
MQAPsingle	4.194	0.631	0.826	1438
ProQ2clust2	4.424	0.594	0.841	1258
MQAPmulti2	4.634	0.637	0.831	1437
ModFOLD4_single	4.710	0.620	0.848	1437
ModFOLD4	4.714	0.637	0**.857**	1437
Pcons-net	4.856	0.615	0.852	1398
ModFOLDclust2	4.986	0.628	0.852	1437
ProQ2clust	5.001	0.596	0.843	1435
MULTICOM-REFINE	5.119	0.611	0.816	1438

Table S4. Comparison between ResQ and other MQAP methods for the local structure quality prediction on the Stage-2 CASP10 MQAP experiment. At Stage-2, up to 150 models for each target, including those being redundant, were included. N_m is the total number of models that a predictor submitted. Best scores are highlighted in bold in each category.

Method	Δd	PCC	AUC	N_m
ResQ	**3.433**	**0.677**	0.865	10800
MULTICOM-REFINE	3.773	0.630	0.839	10800
ModFOLDclust2	3.859	0.668	0.876	10788
ModFOLD4	3.872	0.658	0.871	10788
MQAPmulti	3.911	0.632	0.840	9900
MQAPmulti2	3.928	0.631	0.843	9900
ProQ2clust	3.938	0.643	0.869	10786
MQAPfrag2	3.957	0.638	0.847	9900
MQAPsingle2	3.957	0.638	0.847	9900
ModFOLD4_single	3.998	0.600	0.844	10788
Pcomb	4.010	0.655	**0.877**	10788

Results of B-factor prediction

Three approaches of ResQ were tested to generate B-factor predictions. The template-based prediction is generated by transferring the B-factors of the template proteins as detected by threading, while the profile-based prediction is by training the BFP data on the sequence profile generated from the

PSI-BLAST search. The third combination-based approach is to train the BFP by a combination of both threading template and sequence profiles. A summary of the PCC and AUC between the observed and predicted B-factors by the three approaches are listed in Table S5.

The profile-based approach generated a slightly higher PCC value (0.59) than the template-based approach (0.54), while the combination of the threading templates and sequence profiles achieves the highest PCC (0.61). The difference between the two methods (profile-based and combined) is statistically significant with the p-value of the student t-test below 10^{-12}. A similar tendency is followed by the AUC assessment, where the combined prediction outperforms both template- or profile-based prediction methods.

Table S5. Summary of the B-factor predictions on the 635 test proteins.

Approach	PCC	AUC
Template-based assignment	0.54	0.768
Profile-based training	0.59	0.785
Combination of both	0.61	0.793

REFERENCES

1. Yang, J., Wang, Y. and Zhang, Y. (2015) ResQ: Approach to unified estimation of B-factor and residue-specific error in protein structure prediction. Submitted.
2. Rose, P.W., Beran, B., Bi, C., Bluhm, W.F., Dimitropoulos, D., Goodsell, D.S., Prlic, A., Quesada, M., Quinn, G.B., Westbrook, J.D. *et al.* (2011) The RCSB Protein Data Bank: redesigned web site and web services. *Nucleic Acids Res*, **39**, D392-401.
3. Wu, S.T. and Zhang, Y. (2007) LOMETS: A local meta-threading-server for protein structure prediction. *Nucl. Acids. Res.*, **35**, 3375-3382.
4. Zhang, Y. (2014) Interplay of I-TASSER and QUARK for template-based and ab initio protein structure prediction in CASP10. *Proteins*, **82 Suppl 2**, 175-187.
5. Xu, D., Zhang, J., Roy, A. and Zhang, Y. (2011) Automated protein structure modeling in CASP9 by I-TASSER pipeline combined with QUARK-based ab initio folding and FG-MD-based structure refinement. *Proteins*, **79 (Suppl 10)**, 147-160.
6. Kryshtafovych, A., Barbato, A., Fidelis, K., Monastyrskyy, B., Schwede, T. and Tramontano, A. (2014) Assessment of the assessment: evaluation of the model quality estimates in CASP10. *Proteins*, **82 Suppl 2**, 112-126.
7. Yuan, Z., Bailey, T.L. and Teasdale, R.D. (2005) Prediction of protein B-factor profiles. *Proteins*, **58**, 905-912.
8. Schlessinger, A., Yachdav, G. and Rost, B. (2006) PROFbval: predict flexible and rigid residues in proteins. *Bioinformatics*, **22**, 891-893.
9. Zhang, Y. (2008) I-TASSER server for protein 3D structure prediction. *BMC bioinformatics*, **9**, 40.
